# Driving the precision medicine highway: community health workers and patient navigators

**DOI:** 10.1186/s12967-019-1826-2

**Published:** 2019-03-15

**Authors:** Irma N. Ramos, Kristie N. Ramos, Kenneth S. Ramos

**Affiliations:** 1 Department of Health Promotion Sciences, University of Arizona, Mel and Enid Zuckerman College of Public Health, Tuscon, USA; 2 Department of Medicine, Division of Pulmonary, Allergy, Critical Care and Sleep Medicine, University of Arizona College of Medicine–Tucson, Tucson, AZ 85721 USA; 3 Department of Medicine, Division of Clinical Data Analytics and Decision Support, University of Arizona College of Medicine–Phoenix, Phoenix, USA

**Keywords:** Community health workers, Patient navigators, Precision medicine, Health literay

## Abstract

The general public is currently bombarded with direct-to-consumer advertising, real time “medical” guidance through the internet, access to digital devices that capture health information, and science-based adds that promote foods, cosmetics, and dietary supplements. Unfortunately, much of this information relies on terminology and concepts not well-understood by consumers, particularly those with lower levels of health and genomic literacy. Such constraints align with the limitations of the American public to obtain and process the basic medical information needed to make appropriate healthcare decisions. Low levels of health and genomic literacy render the American public ill-equipped to make informed decisions, use and interpret genomic information, or appreciate the benefits afforded by genomics-based technologies. We propose that coordinated expansion of the roles of community health workers and patient navigators within the precision medicine space can be effectively used to disseminate the knowledge required for the public to benefit from precision medicine advances in healthcare. A well-organized and trained community health worker and patient navigator workforce will provide a voice for the disadvantaged, especially among recent immigrants likely to be experiencing social isolation, language barriers, and economic deprivation. Armed with this knowledge, community health workers and patient navigators can advance the precision medicine agenda and empower disadvantaged communities to take advantage of major advances in the precision medicine era.

## Erratic data capture poses threats to the advancement of precision medicine

Transformative insights in medical knowledge have redefined the way practitioners regard disease, operationalize treatments, and monitor health. This evolution has been contextualized in the form of precision medicine—a platform of healthcare delivery now utilized by various healthcare systems across the nation. Precision Medicine operates on the principle that “more precise” healthcare generates a more meaningful representation of the patient and improves quality of care at reduced costs. Specific examples of precision medicine applications are documented throughout the scientific literature, and best exemplified in clinical oncology [1].

Despite the traditional attainment of personal and family histories during clinical encounters, the reality is that efforts to capture the true contributions of genetics, lifestyle and environment in regards to disease is erratic. Instead, integration of “complementary” data is often incomplete and poorly stratified. Such gaps are impactful in the primary care setting, where the most immediate patient needs are given highest priority, as well as in the inpatient setting, where providers are focused on acute conditions rather than personalization of care. Irrespective of clinical setting, we posit that the benefits of precision medicine cannot be fully realized unless specific strategies are put in place for both patients and practitioners to contextualize, interpret, and utilize precision healthcare.

Advancement of precision medicine reinforces the notion that evidence-based medicine and its footing on randomized clinical trials has disappointingly excluded large segments of the population, particularly individuals of minority descent or those residing in rural communities. On average, there are 1.8 clinical geneticists per one million people in the US [2], and these clinicians are often disproportionately located in large medical centers within highly populated urban areas. A national survey conducted by Shields et al. [3]. notes that minority-serving physicians were significantly less likely to have ever ordered genetic testing to screen for breast cancer, colon cancer, or Huntington’s disease in comparison to physicians who served a fewer number of minority patients. Furthermore, this group reported that minority-serving physicians were significantly less likely to have ever referred a patient for genetic testing or services [3]. Of importance is that those excluded are the very same groups for whom genomic data are not readily available, and for whom lifestyle and environmental determinants have crucial implications. As such, it is not surprising that those underrepresented in clinical research are often those who do not respond as expected to treatment, experience more adverse drug reactions, and often navigate the system without finding appropriate medical solutions [4]. Thus, the one-size-fits-all approach that currently dominates medical practice has become increasingly unfit to meet the needs of a rapidly evolving system of healthcare. We propose that coordinated efforts to expand the roles of community health workers (CHW) and patient navigators (PN) can offer unparalleled opportunities to disseminate the knowledge required for the public sector to benefit from precision healthcare.

Despite the social and environmental determinants of health that are prominently featured in the public health ecosystem, the lay public (that is, the consumers and drivers of the healthcare system) continues to lag behind in the opportunity to take advantage of precision medicine. Currently, the public is bombarded with direct-to-consumer advertising, real time “medical” guidance through the internet, access to digital devices that capture health information, and “science-based” adds that promote food, cosmetics, and dietary supplements. Unfortunately, much of this information uses terminology that is often misunderstood, particularly by consumers with lower levels of health and genomic literacy. Such constraints mirror their limitations to obtain and process the basic medical information needed to make appropriate healthcare decisions. As such, this renders the lay public ill-equipped to make informed decisions and understand genome-based technologies. For instance, though most individuals with a middle school level of education have rudimentary knowledge of Mendelian inheritance, this knowledge is insufficient to understand the concepts of monogenic inheritance or genetic disorders. These knowledge gaps are accentuated in the context of precision medicine, where concepts of polygenic inheritance and chronicity of disease are prominently featured, and complex patterns of inheritance and epigenetic mechanisms play pivotal roles in disease susceptibility and health outcomes. To bridge these critical gaps, we propose the use of CHWs and PNs.

## Community health workers and patient navigators bridge critical gaps in healthcare

CHWs are community-oriented individuals that are trained to establish impactful connections between the community and healthcare systems, and who function to deliver and disseminate health information and assist in the design and execution of community-based participatory research programs [5]. PNs have similar roles, but are often more directly involved in the inpatient setting and focus more on self-management programs that help patients to better navigate through the healthcare system by breaking literacy barriers, reducing fear, and supporting patient-provider communication [6]. In light of these critical functions, CHWs and PNs are in an ideal position to deliver information regarding precision medicine. However, in order to make this a reality, CHWs and PNs will need to receive required training in the basic concepts of precision medicine in order to convey the impact of this novel medicine to potential patient volunteers.

Numerous states already have formalized accreditation programs with established guidelines for specific CHW training [5], and the Patient Advocate Certification Board is currently in the process of developing a comparable set of recognized credential standards for PN programs [6]. In taking advantage of these efforts, appropriate genomic literacy and precision medicine knowledge can be integrated into a curriculum that can provide adequate content expertise and solidify the engagement and intervention efforts of precision medicine initiatives at the local, regional, and national levels. The educational training of CHWs/PNs should also include web-based and in-person workshops. While in-person workshops require additional time devoted to planning, training and point-of-care delivery, and possibly, participation of clinicians versed in genetics and genomics, the web-based component could be completed on an individualized basis and be updated on a regular basis as new information emerges. Web-based modules can also serve as re-fresher tools for post-training education. A multi-component educational system has been successfully used by the Family Health History Training Program in Texas, and this experience can be used to guide development of the program [7, 8]. Lastly, the implementation of the proposed initiatives should carefully consider the additional workload and staffing needs created by the program. Such considerations are essential to enable CHWs/PNs to embrace their newly proposed roles responsibly, particularly in the context of an already understaffed workforce and the need for partnership with state and local governments.

Future iterations of the program can establish an advanced curriculum in precision medicine developed and validated based on previous tutorials created by the National Human Genomics Research Institute. These modules will need to include additional knowledge and proficiency in genetics and genomics, social and environmental determinants of health, and principles of precision healthcare [9]. Continued reliance on culturally appropriate communication and use of the same language should be pivotal elements of precision healthcare education. This advanced curriculum would also benefit from continuity training to ensure knowledge retention, address knowledge gaps, and verify maintenance of certification. A well-organized and trained CHW and PN workforce could be monumental in its ability to provide a voice for the disadvantaged, particularly among recent immigrants likely to experience social isolation, language barriers, and economic deprivation. Armed with knowledge, CHWs and PNs have tremendous potential to advance the precision medicine agenda and empower underserved communities.

## A path forward: community health worker and patient navigator training in precision medicine

Based on the unique position that CHWs and PNs hold within the public health pyramid, these individuals are ideal professionals to address the ongoing gaps in health literacy, and to empower members of the community to make informed decisions about precision-based management and novel therapies. Furthermore, CHWs and PNs can bridge the gap between practitioners and participants and help to inform the public about the need for participation and engagement in medical research to improve the quality and diversity of the national health database. We reason that the creation of a well-designed and validated CHW/PN program in precision medicine should become a top national priority, and has the unique potential to improve health outcomes and “move the healthcare needle” towards a healthier America.

## Abbreviations

CHWs: community health workers
PNs: patient navigators
